# Co-expression of endoglucanase and cellobiohydrolase from yak rumen in lactic acid bacteria and its preliminary application in whole-plant corn silage fermentation

**DOI:** 10.3389/fmicb.2024.1442797

**Published:** 2024-09-17

**Authors:** Xuerui Wan, Yongjie SunKang, Yijun Chen, Zhao Zhang, Huitian Gou, Yu Xue, Chuan Wang, Yaqin Wei, Yuze Yang

**Affiliations:** ^1^College of Veterinary Medicine, Gansu Agricultural University, Lanzhou, China; ^2^The Beijing Municipal Animal Husbandry Station, Beijing, China; ^3^Shanxi Agricultural University, Taigu, Shanxi, China; ^4^Center for Anaerobic Microbes, Institute of Biology Gansu Academy of Sciences, Lanzhou, China

**Keywords:** cellobiohydrolase, endoglucanase, *Lactococcus lactis*, secretory expression, co-expression, silage fermentation

## Abstract

**Introduction:**

Endoglucanase (EG) and cellobiohydrolase (CBH) which produced by microorganisms, have been widely used in industrial applications.

**Methods:**

In order to construct recombinant bacteria that produce high activity EG and CBH, in this study, *eg* (endoglucanase) and *cbh* (cellobiohydrolase) were cloned from the rumen microbial genome of yak and subsequently expressed independently and co-expressed within *Lactococcus lactis* NZ9000 (*L. lactis* NZ9000).

**Results:**

The recombinant strains *L. lactis* NZ9000/pMG36e-usp45-*cbh* (*L. lactis*-*cbh*), *L. lactis* NZ9000/pMG36e-usp45-*eg* (*L. lactis*-*eg*), and *L. lactis* NZ9000/pMG36e-usp45-*eg*-usp45-*cbh* (*L. lactis*-*eg*-*cbh*) were successfully constructed and demonstrated the ability to secrete EG, CBH, and EG-CBH. The sodium carboxymethyl cellulose activity of the recombinant enzyme EG was the highest, and the regenerated amorphous cellulose (RAC) was the specific substrate of the recombinant enzyme CBH, and EG-CBH. The optimum reaction temperature of the recombinant enzyme CBH was 60°C, while the recombinant enzymes EG and EG-CBH were tolerant to higher temperatures (80°C). The optimum reaction pH of EG, CBH, and EG-CBH was 6.0. Mn^2+^, Fe^2+^, Cu^2+^, and Co^2+^ could promote the activity of CBH. Similarly, Fe^2+^, Ba^2+^, and higher concentrations of Ca^2+^, Cu^2+^, and Co^2+^ could promote the activity of EG-CBH. The addition of engineered strains to whole-plant corn silage improved the nutritional quality of the feed, with the lowest pH, acid detergent fiber (ADF), and neutral detergent fiber (NDF) contents observed in silage from the *L. lactis-eg* group (*p* < 0.05), and the lowest ammonia nitrogen (NH_3_-N), and highest lactic acid (LA) and crude protein (CP) contents in silage from the *L. lactis-eg* + *L. lactis-cbh* group (*p* < 0.05), while the silage quality in the *L. lactis*-*cbh* group was not satisfactory.

**Discussion:**

Consequently, the recombinant strains *L. lactis-cbh*, *L. lactis-eg*, and *L. lactis-eg-cbh* were successfully constructed, which could successfully expressed EG, CBH, and EG-CBH. *L. lactis-eg* promoted silage fermentation by degrading cellulose to produce sugar, enabling the secretory expression of EG, CBH, and EG-CBH for potential industrial applications in cellulose degradation.

## 1 Introduction

Cellulase is a complex enzyme system involving a variety of hydrolases. Cellobiohydrolase (exoglucanase), endo-glucanase, and β-glucosidase (cellobiase) work in a coordinated manner to degrade cellulose into small molecules such as glucose that can be absorbed and utilized by the animal intestine (Segato et al., 2014). Cellulases are naturally found in lower animals, insects, bacteria, fungi, plants, and the large intestine and rumen of ruminants (Himmel et al., 2010). The structure and enzymatic activity of cellulases from different sources vary greatly. To construct engineered bacteria with high enzymatic activity, it is necessary first to screen to obtain select genes that express enzyme with excellent activities. The rumen is inhabited by a complex and diverse microbial community and is a natural system that is highly efficient in the conversion and utilization of cellulosic substances. The microbial community metabolizes and efficiently degrades grazing to provide the host with energy and nutrients for survival (Huws et al., 2018), making the rumen a habitat for highly active lignocellulose hydrolases (An et al., 2005; Wei et al., 2016). Sequenced and analyzed the DNA from bovine rumen microorganisms and identified 27,755 putative carbohydrate-active genes, with 57% being enzymatically active against cellulose substrates (Hess et al., 2011). Tiwari et al. (2021) investigations had revealed that over 99% of prokaryotic microorganisms remained elusive through existing pure culture isolation techniques. Directly cloning genes sequences from metagenome of yak rumen microorganisms would be a fast and potent avenue to access cellulase genes.

The cellulase-expressing systems mainly include bacteria, yeast, and plants; each with its own advantages and disadvantages (Garvey et al., 2013). The expression of multiple protein enzymes with complementary activities, preferably in a single strain, is essential for the development of engineered strains that degrade complex substrates (Tarraran and Mazzoli, 2018). The genes expressing endoglucanase and cellobiohydrolase were co-expressed with higher cellulose degradation and biodegradation activities *in Trichoderma reesei* (*T. reevi*) than those obtained from the expression of a single enzyme gene (Wang et al., 2014). The *egl* II (endoglucanase II) and *cbh* II (cellobiohydrolase II) genes from *T. reevi* were co-expressed in *Pichia pastoris* and had the same catalytic properties as the enzymes produced separately (de Amorim Araújo et al., 2015). *Lactococcus lactis* (*L. lactis*), a microorganism with probiotic properties, is usually considered safe for expressing heterologous genes (Zegers et al., 1999; Gaya et al., 2020). Some cellulase genes have been successfully expressed in *L. lactis* to obtain highly active enzymes, which had a widely application (Liu et al., 2005; Ozkose et al., 2009; Zhou et al., 2015). However, the expression level and activity of enzyme proteins in engineered bacteria are still the bottleneck limiting the industrial application of cellulase. Therefore, more attempts are needed, such as selecting more potential enzyme genes when constructing engineered bacteria and co-expressing enzyme genes from different sources in the same expression system.

Silage used lactic acid bacteria to ferment carbohydrates in raw materials to produce organic acids, reduce the pH value of the feed system, inhibit the propagation of harmful microorganisms in the feed, and thus prevent feed deterioration (Fabiszewska et al., 2019). Adding lactic acid bacteria to whole-plant corn silage could enhance the fermentation efficiency and improve the silage quality (Guo et al., 2019). When fermentation substrate was insufficient, fermentation efficiency would be reduced. Adding cellulase could degrade lignocellulose in silage raw materials into monosaccharides, providing more substrates to promote lactic acid bacteria fermentation (Liu et al., 2024; Zhao et al., 2024). Therefore, lactic acid bacteria and cellulase are the two most commonly used silage additives. A large number of studies have focused on the addition of lactic acid bacteria and cellulase in silage alone or in combination to improve the quality of silage (Du et al., 2023, Yu et al., 2024). However, the cost of cellulase hampers its extensive use as feed supplement. Recombinant lactic acid bacteria expressing cellulase can be applied to silage to achieve the effect of adding lactic acid bacteria and cellulase together. Moreover, it can secrete cellulase continuously with the reproduction of recombinant lactic acid bacteria, and exert the synergistic effect of lactic acid bacteria and cellulase. The effects of transgenically engineered *L. lactis* strains on enhancing the conversion of lignocellulose to sugars were better than the combination of cellulase and wild-type *L. lactis subsp* (Liu et al., 2019). At present, there are few reports on the application of recombinant lactic acid bacteria expressing cellulase in silage fermentation (Rossi et al., 2001; Guo et al., 2019). It is likely to be a hotspot of research on silage additives in the future.

The microbial community in yak rumen produced a large number of highly active lignocellulose hydrolases (An et al., 2005; Wei et al., 2016). In previous studies, we expressed β-glucosidase (*bg*) in *Lactococcus lactis* NZ9000 (*L. lactis* NZ9000) from the yak rumen (Wang et al., 2023). Cellulase has been widely used in many industrial applications. To develop new strategies to minimize enzyme production costs, in this study, *cbh* and *eg* genes from metagenome of rumen microorganisms from yak were cloned in the lactobacillus food-grade expression vector. Ribosome binding sites (RBS) were introduced before each gene to prepare a secretion-type expression vector and transformed into *L. lactis* NZ9000 through electroporation to obtain the recombinant bacteria co-expressing cellobiohydrolase and endoglucanase. The recombinant enzymatic activity and optimal growth conditions of the recombinant strains were analyzed. Subsequently, the recombinant strains were added to whole-plant corn for laboratory silage, and those roles in the fermentation of silage were assessed. This study laid a foundation for the application of lignocellulose hydrolases and recombinant LAB in feed preparation.

## 2 Materials and methods

### 2.1 Bacterial strains, plasmids, and culture conditions

Table 1 is the bacterial strains and plasmids for this study. Luria-Bertani (LB) medium (both agar and broth) was used to culture *Escherichia coli* DH5α at 37°C, and GM17 medium was used to culture *L. lactis* NZ9000 grown in at 30°C. Erythromycin (0.1 μg/mL; Solarbio Science & Technology Co., Ltd., Beijing, China) was utilized used to screen the positive recombinants of *L. lactis* NZ9000 with the pMG36e backbone.

**Table 1:** Bacterial strains and plasmids used in this study.

Strain and plasmid		Relevant trait(s)	Source or reference
Strains	*Escherichia coli* DH5α	supE44 Δlac U169 (Φ80 lacZ ΔM15) hsdR17 recA1, endA1 gyrA96 thi-l relA1	This laboratory
	*L. lactis* NZ9000	Express recombinant plasmids	Kuipers et al. (1998)
	*E. coli* DH5α/pMG36e*-*usp45*-eg*	*E. coli* DH5α with pMG36e-usp45-*eg*	This study
	*E. coli* DH5α*/*pMG36e*-*usp45*-cbh*	*E. coli* DH5α with pMG36e*-*usp45*-cbh*	This study
	*E. coli* DH5α*/*pMG36e*-*usp45*-eg-*usp45*-cbh*	*E. coli* DH5α with pMG36e*-*usp45*-eg-*usp45*-cbh*	This study
	*L. lactis* NZ9000*/*pMG36e	*L. lactis* NZ9000 with pMG36e	This study
	*L. lactis* NZ9000*/*pMG36e*-*usp45*-eg* (*L. lactis-eg*)	*L. lactis* NZ9000 with secretory expression EG	This study
	*L. lactis* NZ9000*/*pMG36e*-*usp45*-cbh* (*L. lactis-cbh*)	*L. lactis* NZ9000 with secretory expression CBH	This study
	*L. lactis* NZ9000*/*pMG36e*-*usp4*5-eg-*usp45*-cbh* (*L. lactis- eg -cbh*)	*L. lactis* NZ9000 with secretory expression EG and CBH	This study
Plasmids	pMG36e	Emr; expression vector with the P32 promoter, multiple cloning sites (MCF) and prtP translational terminator	van de Guchte et al. (1989)
	pMG36e-usp45-*eg*	Emr; secretory expression of *eg*	This study
	pMG36e-usp45-*cbh*	Emr; secretory expression of *cbh*	This study
	pMG36e-usp45-*eg*-usp45-*cbh*	Em^r^; secretory expression of *eg* and *cbh*	This study

### 2.2 Plasmid recombination

The primers designed based on NCBI *eg* and *cbh* sequences are provided in Table 2. Twenty 5–6 year-old gibbed and male Tianzhu yaks were used to extract the whole genome from their yak rumen fluid using the Cetyltrimethylammonium Bromide (CTAB) methods. Each yak which weighed about 250 kg was raised on wild grass at Wushaoling Pasture in the Tibetan Autonomous County of Tianzhu (Gansu Province, China) (Popova et al., 2010; Wang et al., 2023). *cbh*-F/R and *eg*-F/R were used as primers to amplify the *cbh* and *eg* by PCR. PCR was conducted in a total volume of 50 μL, which contained the following items: EasyPfu Polymerase buffer, 2 μL (TransGen Biotech Co., Ltd. Beijing, China); 10 × EasyPfu buffer 5 μL; 2.5 mmol/L dNTPs, 5 μL; the whole genome of yak rumen, 2 μL; nuclease-free water, 26 μL; each PCR primer 2.5 μL. The operation was carried out as follows: pre-denaturation at 95°C for 5 min and 30 cycles of 95°C for 30 s, 56.5°C for 30 s, 72°C for 90 s, and 72°C extension for 10 min (Wang et al., 2023). Easy Pure PCR Purification Kit (TransGen Biotech Co., Ltd., Beijing, China) was used to purify the PCR products of *cbh* and *eg*.

**Table 2:** cbh and e*g* amplification primers.

Primer	Sequence (5′–3′)	Restriction site
*cbh*-F	GCTCTAGAAAGAAGGAGATATACATGCAAAAAAAAGATTATCTCAGCTACAGCAGGTCGGTACTTCCCA	*Xba* I
*cbh*-R	CCCAAGCTTTACAGGCACTGAGAGTAATA	*Hind* III
*eg*-F	CGAGCTCAAGAAGGAGATATACATGCAAAAAAAAGATTATCTCAGCTAATGAAACGGTCAATCTCTA	*Sac* I
*eg*-R	GCTCTAGACTAATTTGGTTCTGTTCCCCA	*Xba* I

The single underlines were the enzyme cut sites; the wavy line was RBS; the double underline for usp45 signal peptide.

The purified *eg* and *cbh* PCR productswere digested with *Sac*I/*Xba* Iand *Xba* I/*Pst* I (TakaRa Biotechnology Co., Ltd. Dalian, China), respectively, which was then linked with the digested amplicon using T4-DNA ligase (TakaRa Biotechnology Co., Ltd. Dalian, China) to produce the recombinant vectors pMG36e-usp45-*eg* and pMG36e-usp45-*cbh*. Subsequently, the purified *eg* PCR products and pMG36e-usp45-*cbh* were, respectively, digested with *Sac*Iand *Xba* I, and the digestion product of *eg* and pMG36e-usp45-cbh were linked using T4-DNA ligase to produce the recombinant vectors pMG36e-usp45-*eg*-usp45-*cbh which were* the introduced into *E. coli* DH5α. The positive recombinants of *E. coli* DH5α/pMG36e-usp45-eg, *E.coli* DH5α/pMG36e-usp45-*cbh*, and *E. coli* DH5α/pMG36e-usp45-*eg*-usp45-*cbh* were verified by PCR and double enzymes digestion (Wang et al., 2023). After the verification, the correct recombinant plasmids were selected for sequencing, the results of which were compared with NCBI Basic Local Alignment Search Tool (BLAST).

### 2.3 Transformation of the recombinant plasmid

A Gene Pulser Xcell (Times-Legend Bioscientific Co., Ltd.) was used to transform the verified correct recombinant plasmids into the *L. lactis* NZ9000 via electroporation under the following conditions: at a pulse voltage of 2,200 V, pulse resistance of 100 Ω, pulse capacitance of 25 μF, and a pulse time of 3 s. The verified correct recombinant plasmids included pMG36e-usp45-*cbh*, pMG36e-usp45-*eg*, and pMG36e-usp45-*eg*-usp45-*cbh.* 0.1 μg/mL erythromycin was used to select the positive transformants of *L. lactis*/pMG36e-usp45-*eg* (*L. lactis-eg*), *L. lactis*/pMG36e-usp45-*cbh* (*L. lactis*-*cbh*), *L. lactis*/pMG36e-usp45-*eg*-usp45-*cbh* (*L. lactis-eg*-*cbh*), which were then verified by PCR (Wang et al., 2023).

### 2.4 Enzyme assays and protein analysis of recombinant *Lactococcus lactis* NZ9000

The inoculum of *L. lactis-eg*, *L. lactis*-*cbh*, and *L. lactis-eg*-*cbh* were added to the sample in GM17 plates whichcontained 1 g/L of CMC-Na and 0.1 μg/mL of erythromycin (pH 7.0) to culture for 48 h at 30°C. After that, they were exposed to 1 g/L Congo red solution. Then the plates were incubated for 30 min and washed with 1 mol/L NaCl to discover the clear zones against a red background which developed via hydrolysis of CMC-Na (Wang et al., 2023).

The engineered strains were incubated at 30°C for 36 h to collect the supernatant. The precipitation method (An et al., 2005; Niu et al., 2018) was used to purify recombinant proteins from the supernatant. By sodium dodecyl sulfate–polyacrylamide gel electrophoresis (SDS-PAGE), the molecular masses of the enzymes of recombinant were estimated. By measuring the amount of reduced sugars released by recombinant enzymes using the 3,5-dinitrosalicylic acid method, the activity of all enzymes was determined with Glucose as a standard (Geem et al., 2021). Regenerated amorphous cellulose (RAC), CMC-Na, dried cotton, microcrystalline cellulose (MCC), filter paper, and acid were used as substrates to test the cellulase activity of individual and recombinant fusion enzymes, and recombinant proteins inactivated by boiling inactivation were used as blank controls (Zhao et al., 2021).

### 2.5 Enzyme assays of recombinant at temperature and pH

To measure the optimum reaction temperature for the recombinant enzyme, the supernatant of the recombinant enzyme was used to react with the optimum substrate at different temperatures (30–90°C) for 30 min. To measure the optimum pH for recombinase activity, the recombinase supernatant in several buffers of different pH (pH 3.0–9.0) was sued to react with the optimum substrate at the optimum temperature for 30 min to determine (Wang et al., 2023).

### 2.6 Enzyme kinetic parameter assays

Regenerated amorphous cellulose at various concentrations (0.1–10 nM) was used as the substrate to determine the activity of recombinant enzymes at 80°C and pH 6.0. The data were fitted in GraphPad Prism 8.4.2 with Michaelis–Menten equation fitting and Lineweaver-Burk plots (double-reciprocal plots) to estimate the enzyme kinetic parameters, such as maximum enzymatic activity for substrate conversion (V_max_), Michaelis–Menten constant (K_m_), turnover number (K_cat_), and K_cat_/K_m_.

### 2.7 Enzyme assays at different metal ion concentrations

To ensure the final concentrations of the solutions reached 1 mmol/mL and 5 mmol/mL, 1% RAC was used as substrate at 80°C and pH 6.0, and K^+^, Mg^2+^, such elements as Mn^2+^, Zn^2+^, Hg^2+^, Fe^2+^, Co^2+^, Ca^2+^, and Cu^2+^ were added to the reconstituted enzyme supernatant and optimal substrate for the reaction. To ensure the final concentrations of the solutions were 1 and 10% at optimal temperature and pH for 30 min, Tween20 and Tween80 were added to the reconstituted enzyme supernatant and optimal substrate for the reaction. Different concentrations of ions and solutions affected the activity of the recombinant enzyme. The effects were measured and compared to the solutions without metal ions and chemical reagents used as blank controls (Wang et al., 2023).

### 2.8 Laboratory study of whole-plant corn silage with added recombinant *Lactococcus lactis* NZ9000

Recombinant *L. lactis* NZ9000 were added to whole-plant corn silage in polyethylene bags in the laboratory. The experiment included six treatments, namely, *L. lactis-eg*, *L. lactis-cbh*, *L. lactis-eg-cbh*, *L. lactis-eg* + *L. lactis-cbh*, *L. lactis* NZ9000, and GM17 liquid medium as the control. Recombinant *L. lactis* NZ9000 was cultured in GM17 liquid medium to the logarithmic growth stage, after which the concentration of the culture was adjusted to 1 ×10^9^ CFU/mL and the bacteria were added to whole-plant corn with a 70% water content and 2–4 cm at a ratio of 5 mL/kg, and mixed evenly. The evenly mixed silage raw materials were then packed into polyethylene bags (500 g/bag) and compacted, after which the silage bags were sealed using a vacuum sealer. Each treatment was repeated three times. The sealed silage bags were placed in a constant temperature environment (20°C) for fermentation. On day 30, samples were taken from the silage and assessed by measuring their pH values, the levels of water soluble carbohydrate (WSC), neutral detergent fiber (NDF), acid detergent fiber (ADF), crude protein (CP), lactic acid (LA), acetic acid (AA), and ammonia nitrogen (NH_3_-N) (Wan et al., 2016).

### 2.9 Data statistics and analyses

To assess how varied temperatures, pH, ions, and solution concentrations affected the enzymatic activity of the recombinant enzymes, GraphPad Prism 9.5 two-way ANOVA was used to analyze the data. The means were compared using Tukey’s test for significance at *p* < 0.05. To evaluate the influence of recombinant *L. lactis* NZ9000 on the quality of whole-plant corn silage, the data were statistically analyzed using SPSS 18.0, and the mean values were compared by Duncan method for significance at *p* < 0.05.

## 3 Results

### 3.1 Construction and identification of recombinant plasmids

**Figure 1:**
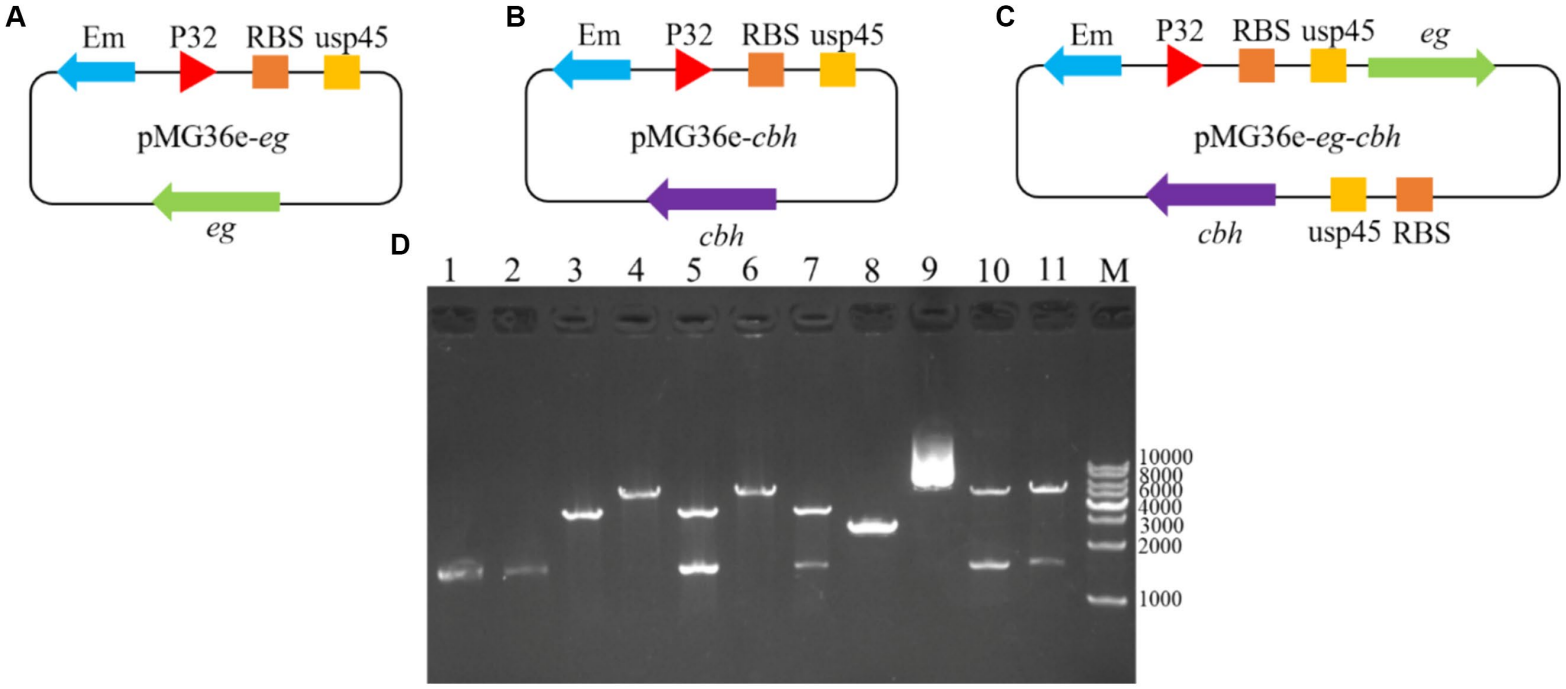
Recombinant plasmids construction strategy and double enzyme digestion verification. **(A)** Recombinant plasmid construction strategy of pMG36e-usp45-*eg*; **(B)** Recombinant plasmid construction strategy of pMG36e-usp45-*cbh*; **(C)** Recombinant plasmid construction strategy of pMG36e-usp45-*eg*-usp45-*cbh*; **(D)** lane 1: Colony PCR of eg; lane 2: Colony PCR of *cbh*; lane 3: Single enzyme digestion of empty vector pMG36e; lane 4: Single enzyme digestion of pMG36e-usp45-*eg*; lane 5: Double enzyme digestion of pMG36e-usp45-*eg* use SacIand *XbaI*; lane 6: Single enzyme digestion of pMG36e-usp45-*cbh*; lane 7: Double enzyme digestion of pMG36e-usp45-*eg* use *Xba I* and *Pst I*; lane 8: Colony PCR of *eg-cbh*; lane 9: Single enzyme digestion of pMG36e-usp45-*eg*-usp45-*cbh*; lane 10: Double enzyme digestion of pMG36e-usp45-*eg*-usp45-*cbh* use *SacI*and *XbaI*; lane 11: Double enzyme digestion of pMG36e-usp45-*eg*-usp45-*cbh* use *Xba I* and *Pst I*; M: DL10000 DNA Marker.

**Figure 2:**
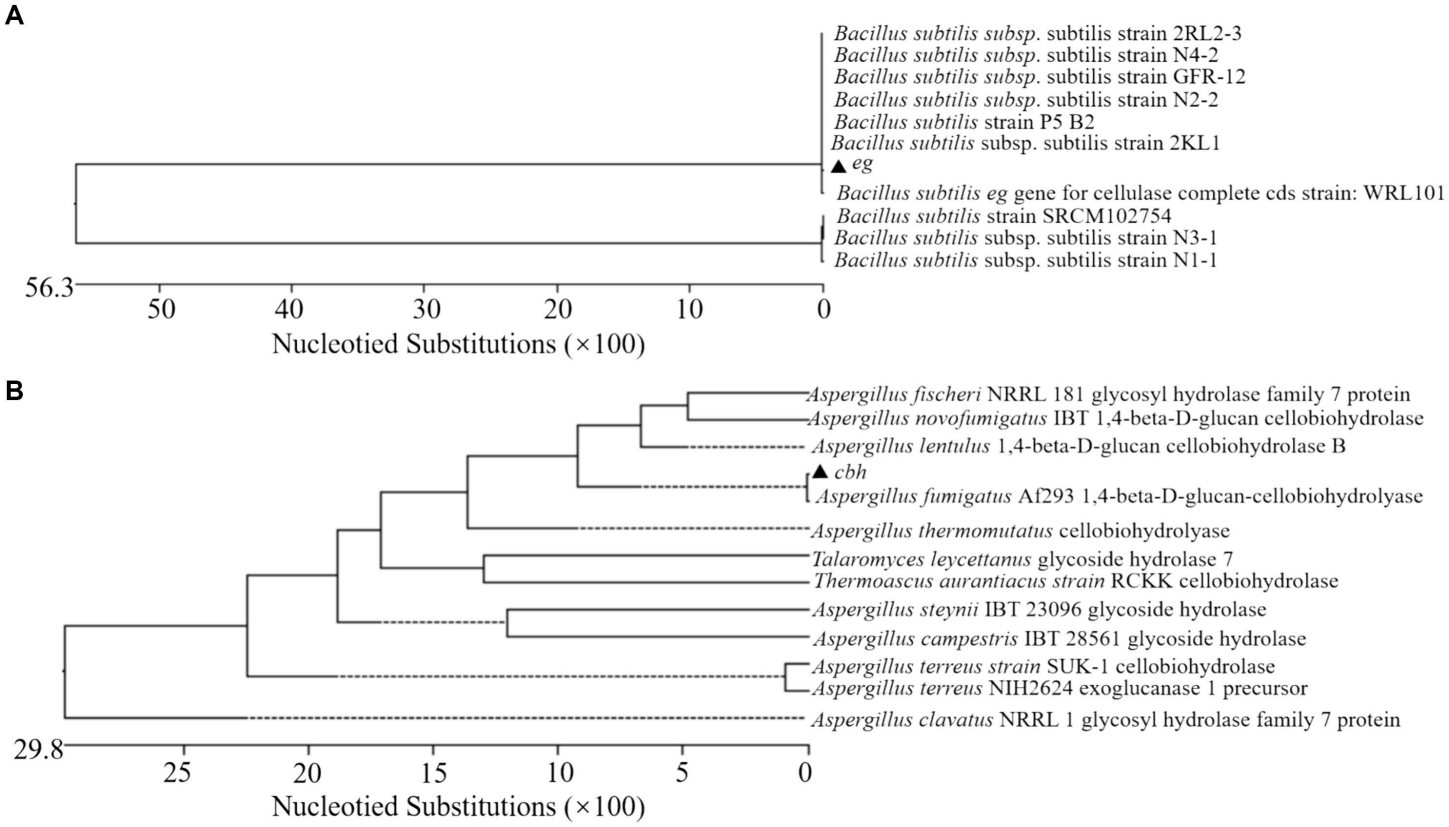
Homology analysis and evolutionary tree construction of *eg* **(A)** and *cbh* **(B)**.

Three recombinant plasmid maps designed for this study are shown in Figure 1. The recombinant plasmid contained the ribosomal binding site (RBS), the signal peptide usp45, and the promoter P32. pMG36e-usp45-*eg*, pMG36e-usp45-*cbh*, and pMG36e-usp45-*eg*-usp45-*cbh* were successfully constructed and verified via double digestion. Sequencing showed the size of *eg* and *cbh* were 1,500 and 1,599 bp (Figure 1D). We performed BLAST alignment of the obtained *eg* and *cbh* gene sequences in NCBI and then selected sequences with high similarity to perform multiple sequence alignment and genetic evolution by the MegAlign software in DNAstar. The results showed a 100% homology of the *eg* gene with the *eg* of *Bacillus subtilis* (GenBank Accession No. AB695293.1) and a 100% homology of the *cbh* gene with the *cbh* of *Aspergillus fumigatus* (GenBank Accession No. XM_745951.1) (Figure 2).

### 3.2 Enzyme proteins expression of recombinant strains

**Figure 3:**
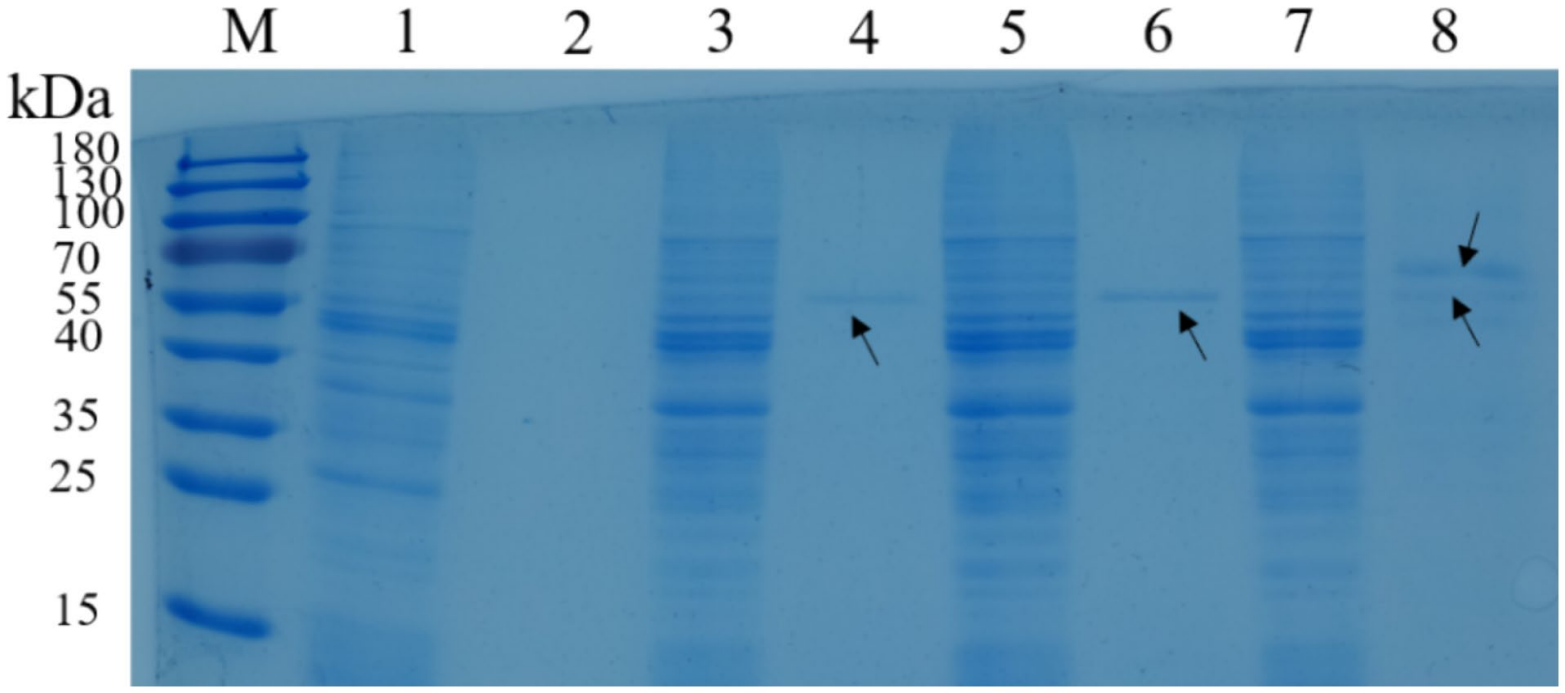
SDS-PAGE of recombinant strains. M: protein molecular weight marker; lane 1: intracellular protein of *Lactococcus lactis* NZ9000; lane 2: supernatant of *L. lactis* NZ9000 via TCA/acetone precipitation; lane 3: intracellular protein of *L. lactis-eg*; lane 4: supernatant of *L. lactis-eg* via TCA/acetone precipitation; lane 5: intracellular protein of *L. lactis-cbh*; lane 6: supernatant of *L. lactis-cbh* via TCA/acetone precipitation; lane 7: intracellular protein of *L. lactis-eg-cbh*; and lane 8: supernatant of *L. lactis-eg-cbh* via TCA/acetone precipitation.

*Lactococcus lactis-eg* and *L. lactis-cbh* could secretory express EG and CBH proteins, respectively. The molecular weights of EG and CBH were 54.9 and 56.4 kDa by analysising use Quantity One 1-D software (version 4.6.2). In addition, via SDS-PAGE showed that *L. lactis-eg-cbh* had two bands of similar size between 55 and 70 kDa, indicating that *L. lactis-eg-cbh* could co-express and secrete recombinant protein EG and CBH (Figure 3).

### 3.3 Enzyme activity of recombinant strains

**Figure 4:**
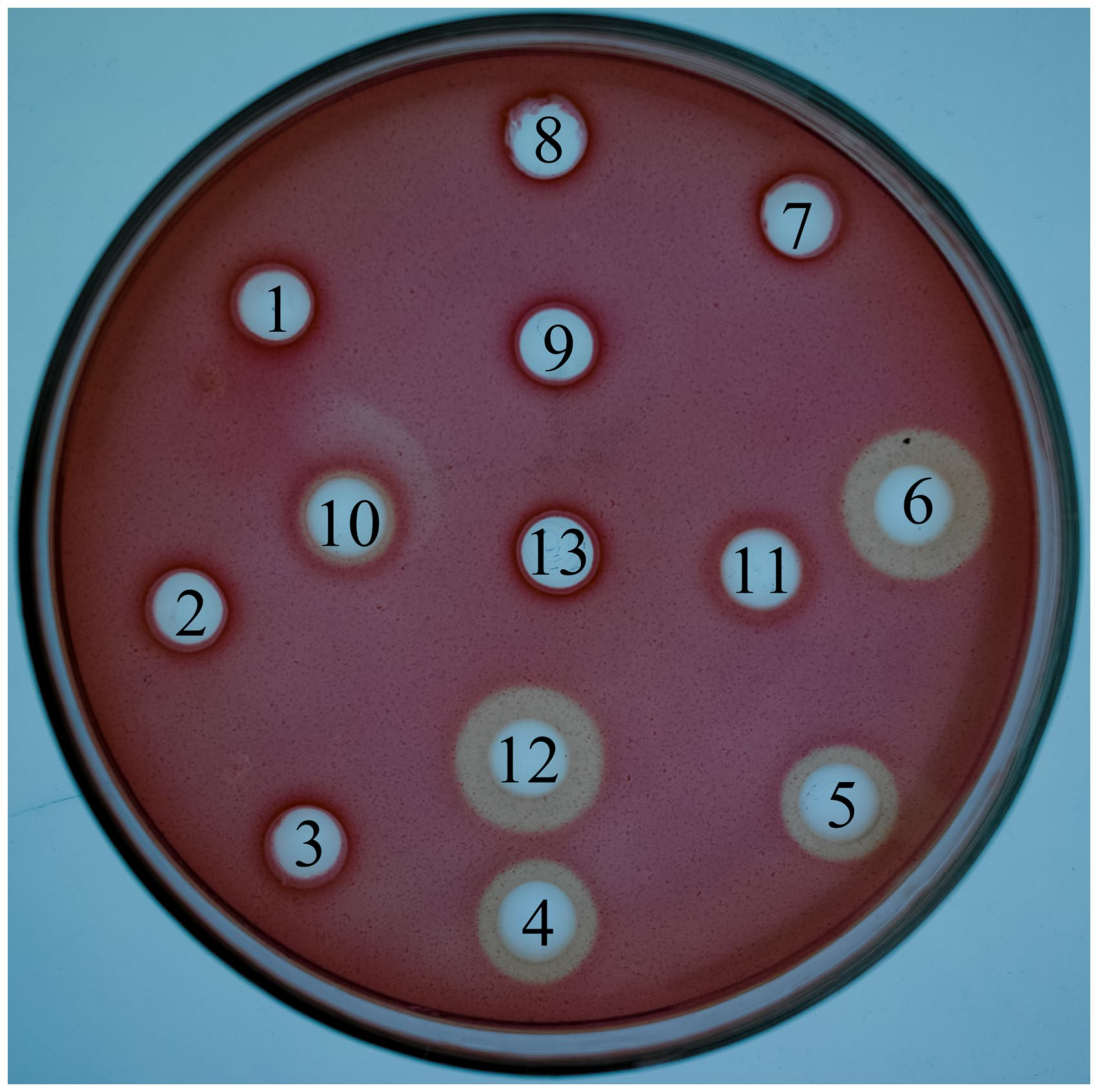
Congo red staining with CMC-Na. 1: Supernatant of *Lactococcus lactis* NZ9000; 2: The bacterial liquid of *L. lactis* NZ9000; 3: Supernatant of *L. lactis* NZ9000 via TCA/acetone precipitation; 4: Supernatant of *L. lactis-eg*; 5: The bacterial liquid of *L. lactis-eg*; 6: Supernatant of *L. lactis-eg* via TCA/acetone precipitation; 7: Supernatant of *L. lactis-cbh*; 8: The bacterial liquid of *L. lactis-cbh*; 9: Supernatant of *L. lactis-cbh* via TCA/acetone precipitation; 10: Supernatant of *L. lactis-eg-cbh*; 11: The bacterial liquid of *L. lactis-eg-cbh*; 12: Supernatant of *L. lactis-eg-cbh* via TCA/acetone precipitation; 13: Blank.

As shown in Figure 4, *L. lactis-eg* had significant hydrolysis circles around them. Conversely, *L. lactis-cbh* did not have hydrolysis circles around them. The supernatant and bacterial liquid of *L. lactis-eg-cb*h showed a slight hydrolysis circle, but the supernatant showed an obvious hydrolysis circle after concentration.

**Figure 5:**
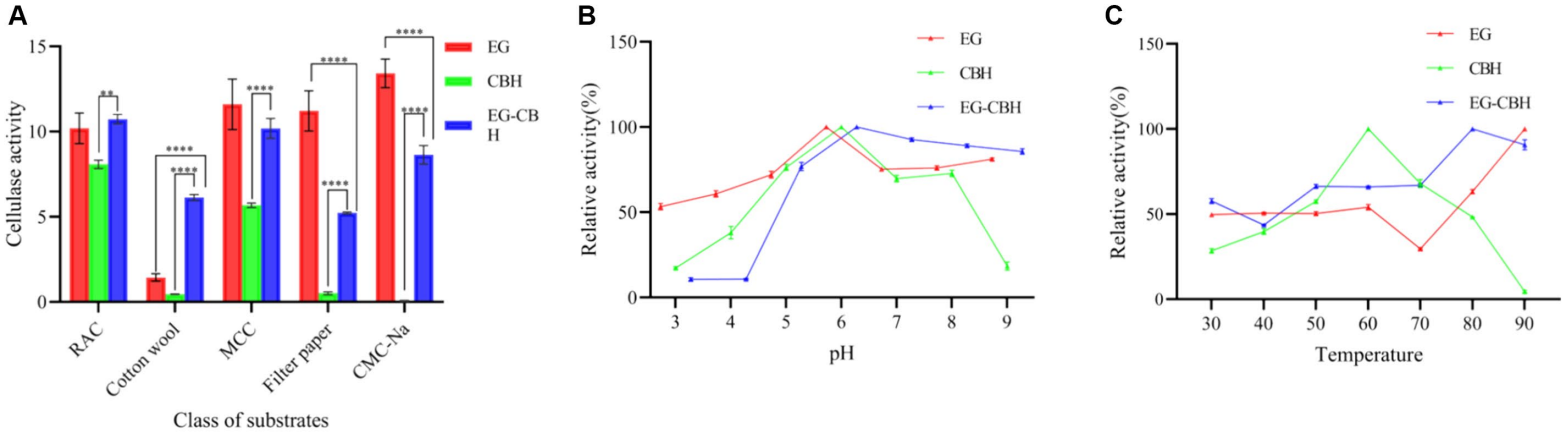
Effect of substrate, pH, and temperature on recombinant enzymes activity. **(A)** Substrate specificity of recombinant enzymes; **(B)** Effect of pH on recombinant enzymes activity; **(C)** Effect of temperature on recombinant enzymes activity; ^*^ represents the difference within experimental groups, ^**^p < 0.01, ^****^p < 0.0001; Error bars in graphs represent standard deviation of the data.

The crude enzyme solution of *L. lactis-eg*, *L. lactis-cbh*, and *L. lactis-eg-cbh* exhibited different substrate specificities. Among them, the activity of recombinant enzyme EG was the highest for CMC-Na, at 13.42 U/mL and the specific substrate of recombinant enzyme CBH was RAC, with the activity being 8.07 U/mL. The co-expression product of EG and CBH had the highest specificity for RAC, with the enzyme activity being 10.73 U/mL. The hydrolytic activity of the co-expression products of EG and CBH on RAC, cotton wool, MCC, filter paper, and CMC-Na was significantly higher than that of CBH expressed singly. Compared with the singly expressed EG, the co-expressed products of EG and CBH had significantly improved the hydrolysis activity on cotton wool but not on RAC and MCC, while the hydrolysis activity on filter paper and CMC-Na was significantly decreased (Figure 5A).

### 3.4 Optimum reaction pH and temperature of recombinant enzymes

The activities of recombinant enzymes EG, CBH, and EG-CBH increased with the increase of pH in the range of 3.0–6.0. The optimal pH range of enzyme activity of EG, CBH, and EG-CBH was 5.0–8.0, and the relative enzyme activity was more than 70%, especially at pH 6.0 (Figure 5B).

Within the range of 30–60°C of reaction temperature, the activity of EG, CBH, and EG-CBH enzymes increased with the temperature, and the activity of CBH enzymes peaked when the temperature was 60°C. When the temperature was higher than 60°C, the enzyme activity of EG decreased briefly at 70°C and then continued to rise. Interestingly, the co-expression product of EG and CBH showed tolerance for higher temperatures, and the enzyme activity of EG-CBH reached its peak at 80°C (Figure 5C).

### 3.5 Determination of substrates on the kinetic parameters of recombinant enzymes

At the optimal temperature and pH, RAC was used as the substrate to assay the kinetic parameters of the recombinant enzymes. Compared with V_max_, K_m_, K_cat_, and K_cat_/K_m_ values of the recombinant enzymes in bacterial supernatant of *L. lactis-eg*, *L. lactis-cbh*, and *L. lactis-eg-cbh*, the results of the assessment were shown in Table 3. The bacterial supernatant of *L. lactis-eg* was characterized as having the highest V_max_ (1,601 μmol∙min^−1^ mg^−1^), K_cat_ (73.09 s^−1^), K_cat_/K_m_ (0.44 L∙s^−1^∙μmol^−1^), and the lowest K_m_ (167.6 μmol∙L^−1^), but bacterial supernatant of *L. lactis-cbh* and *L. lactis-eg-cbh* had very similar V_max_ (1,491 and 1,491 μmol∙min^−1^ mg^−1^), K_m_ (174.6 and 174.6 μmol∙L^−1^), K_cat_ (66.04 and 67.63 s^−1^), and K_cat_/K_m_ (0.38 and 0.39 L∙s^−1^∙μmol^−1^).

**Table 3:** Kinetic parameters of recombinant enzymes.

Strain	*V*_max_ (μmol∙min^−1^ mg^−1^)	*K*_m_ (μmol∙L^−1^)	*K*_cat_ (s^−1^)	*K*_cat_/*K*_m_ (L∙s^−1^∙μmol^−1^)
*L. lactis-eg*	1,601	167.6	73.09	0.44
*L. lactis-cbh*	1,491	174.6	66.04	0.38
*L. lactis-eg-cbh*	1,491	174.6	67.63	0.39

V_max_, Maximum enzymatic activity for substrate conversion; K_m_, Michaelis–Menten constant; K_cat_, Turnover number.

### 3.6 Effect of ions and chemical reagents on recombinant enzymes

Mn^2+^, at 1 mmol/L was found to promote the enzyme activity of CBH. When the concentration of Mn^2+^ increased, it significantly promoted the enzyme activity of EG-CBH, while Mn^2+^ had no effect on the activity of EG. The low concentration of Zn^2+^ significantly inhibited the enzyme activity of EG-CBH, which became zero with the increase in ion concentration, while Zn^2+^ had no effect on the activity of a single recombinant enzyme. Fe^2+^ promoted the activity of the three recombinants, and the high concentration of Fe^2+^ increased the activity of CBH and EG-CBH. Ba^2+^ had no effect on the activity of any single recombinant enzyme and significantly promoted the activity of EG-CBH. Ca^2+^ at 1 mmol/L promoted the activity of EG and inhibited the activity of EG-CBH. When the Ca^2+^ ion concentration increased, the enzyme activity of EG-CBH was promoted. At low concentration, Cu^2+^ only promoted CBH. When the concentration reached 5 mmol/L, it inhibited the EG activity and significantly promoted the CBH and EG-CBH activities.

**Figure 6:**
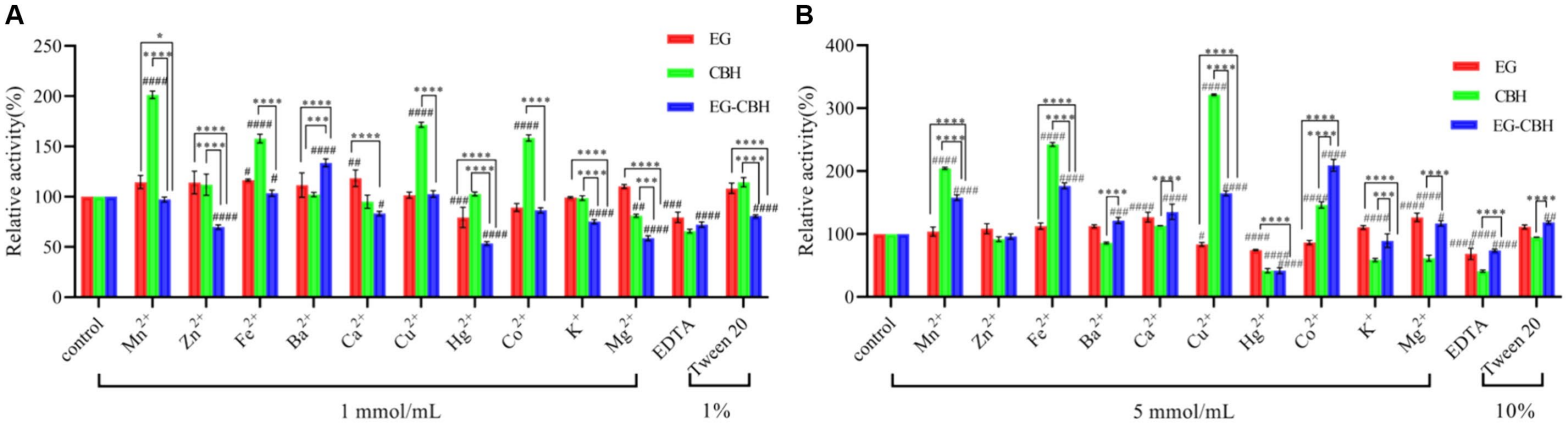
Effect of ions and chemical reagents on recombinant enzymes. **(A)** Effect of ions at and 1% solution on enzyme activity; **(B)** Effect of ions at 5 mmol/mL and 10% solution on enzyme activity (^#^represents the difference between experimental groups and control group, ^#^0.01 < *p* < 0.05, ^##^*p* < 0.01, ^###^*p* < 0.001, ^####^*p* < 0.0001; ^*^ represents the difference within experimental groups, ^*^0.01 < *p* < 0.05, ^***^p < 0.001, ^****^p < 0.0001; Error bars in graphs represent standard deviation of the data).

With the increase in ion concentration, Hg^2+^ significantly inhibited the activity of the three recombinant enzymes. When the concentration of Co^2+^ was 1 mmol/L, it could only promote CBH activity. When the concentration reached 5 mmol/L, it significantly promoted CBH and EG-CBH activities but had no effect on EG activity. At 1 mmol/L, K^+^ inhibited the enzyme activity of EG-CBH, while 5 mmol/L, K^+^ inhibited the enzyme activity of CBH and EG-CBH. At 1 mmol/L, Mg^2+^ inhibited the enzyme activity of CBH, and EG-CBH, and when the concentration increased, it promoted the enzyme activity of EG while inhibiting the activity of CBH, EG-CBH; in addition, the inhibition of EG-CBH was weakened. The inhibitory effect of EDTA on the activities of three enzymes increased with the increase in concentration. Tween had different effects on EG-CBH at different concentrations. At 1% concentration, Tween promoted the activity of the EG-CBH enzyme and produced opposite results with the increase in concentration (Figures 6A,B).

### 3.7 Effect of recombinant strains on the quality of whole-plant corn silage

Recombinant strains were added to the whole corn, and the quality of the silage were assessed on day 30 after the addition; the results of the assessment are shown in Table 4. The pH of silage in the *L. lactis-eg* group was found to be the lowest, followed by the *L. lactis-eg + L. lactis-eg-cbh* group. The pH values were significantly lower in these two groups compared with the other groups (*p* < 0.05), although there was no significant difference between the two groups. The LA content in the *L. lactis-eg + L. lactis-cbh* group was highest, followed by the *L. lactis-eg* group, which were both significantly higher than the contents in other groups (*p* < 0.05). The AA contents of the silage in all recombinant *L. lactis* NZ9000 groups were significantly higher than those in the *L. lactis* NZ9000 and Control groups (*p* < 0.05), with the highest AA content observed in the *L. lactis-eg-cb* group, significantly higher than that in the other three recombinant *L. lactis* NZ9000 groups. The LA/AA ratio in the silage was highest in the *L. lactis-eg + L. lactis-cbh* group, followed by the *L. lactis-eg* group, with the *L. lactis-eg-cbh* group being the lowest. The NH_3_-N content of silage in the *L. lactis-eg + L. lactis-cbh* group was significantly lower than that in other groups (*p* < 0.05), while the WSC content was highest in the *L. lactis-eg* group, followed by the *L. lactis-eg + L. lactis-cbh* and *L. lactis-eg-cbh* groups, significantly higher than that in the other groups (*p* < 0.05). The contents of ADF and NDF in the *L. lactis-eg* group were the lowest, followed by the *L. lactis-eg + L. lactis-cbh* group, differing significantly from those in other groups (*p* < 0.05). The CP content of silage in the *L. lactis-eg + L. lactis-cbh* group was found to be highest, followed by the *L. lactis-eg* group, both of which were significantly higher than that observed in the other groups (*p* < 0.05).

**Table 4:** Effect of recombinant strains on the quality of whole-plant corn silage.

	*L. lactis-eg*	*L. lactis-cbh*	*L. lactis-eg-cbh*	*L. lactis-eg + L. lactis-cbh*	*L. lactis* NZ9000	Control	*p* value
pH	3.70 ± 0.30b	3.92 ± 0.26a	3.85 ± 0.09a	3.75 ± 0.14b	4.09 ± 0.20a	4.12 ± 0.20a	0.02
LA (%DM)	6.25 ± 0.11b	5.38 ± 0.28d	5.62 ± 0.21c	6.71 ± 0.18a	5.44 ± 0.09d	5.33 ± 0.13d	0.02
AA (%DM)	2.11 ± 0.24b	2.05 ± 0.06b	2.52 ± 0.14a	2.04 ± 0.64b	1.83 ± 0.71c	1.81 ± 0.14c	0.03
LA/AA	2.96	2.62	2.23	3.28	2.97	2.94	
NH_3_-N (g/kgDM)	6.66 ± 1.04b	6.91 ± 0.47a	7.09 ± 0.34a	6.47 ± 0.47b	7.15 ± 0.32a	7.11 ± 0.22a	0.03
WSC (%DM)	6.55 ± 0.27a	5.23 ± 0.14b	5.79 ± 0.08a	6.15 ± 0.15b	5.30 ± 0.12b	5.61 ± 0.07ab	0.04
NDF (%DM)	50.68 ± 0.49b	52.43 ± 0.50a	53.22 ± 0.35a	51.20 ± 0.16b	52.14 ± 0.19a	52.57 ± 0.35a	0.01
ADF (%DM)	26.27 ± 0.25c	28.46 ± 0.34a	28.41 ± 0.34a	27.37 ± 0.30b	28.50 ± 0.36a	28.63 ± 0.43a	0.02
CP (%DM)	9.12 ± 0.33a	8.47 ± 0.18b	8.37 ± 0.18b	9.08 ± 0.65a	8.57 ± 0.17b	8.26 ± 0.27b	0.01

DM, Dry matter; LA, Lactic acid; AA, Acetic acid; NH_3_-N, Ammonia nitrogen; WSC, Water soluble carbohydrate; NDF, Neutral detergent fiber; ADF, Acid detergent fiber; CP, Crude protein. The different lowercase letters in the same line mean significant differences at *p* < 0.05.

## 4 Discussion

Cellulose was an important carbon source for microorganisms in yak rumen, and Glycoside hydrolases (GHs) were the most abundant class of carbohydrate-active enzymes (Jiang et al., 2022). Therefore, rumen microorganisms is one of the main sources of cellulase. High throughput 16S rRNA sequencing and metagenomic analysis have indicated that the rumen microbiome of plateau yaks has more enzymes genes involved in cellulase and hemicellulase than that of cattle (Dashtban et al., 2010; Zhao et al., 2022). In addition, cellulase produced by rumen fungi is one of the most active fibrolytic enzymes (Selinger et al., 1996). Therefore, *cbh* and *eg* genes from uncultured rumen microorganisms of yak were cloned in pMG36e to construct expression vectors pMG36e-usp45-*eg*, pMG36e-usp45-*cbh*, and pMG36e-usp45-*eg*-usp45-*cbh*. Engineered strains *L. lactis-eg*, *L. lactis-cbh*, *and L. lactis-eg-cbh* were constructed successfully, and cellobiohydrolase and endoglucanase were successfully independently and co-expressed. pMG36e was constructed based on the transcriptional and translational signals of the protease gene of the *L. lactis* milk lipid subspecies; it was a constitutive expression vector for the inserted gene in *L. lactis* NZ9000 was a manually constructed recombinant host with the nisin-controlled gene expression system, which has advantages over other expression systems for expressing recombinant proteins and has been used widely for expressing a variety of proteins from other bacteria (Liu et al., 2022). Compared with *E. coli*, *L. lactis* has a single outer membrane and is able to secrete heterologous proteins directly into the extracellular environment; moreover, the total protein enzyme expressed in *L. lactis* has been shown to be higher than in *E. coli* (Liu et al., 2017). The ability of *L. lactis* to efficiently express and secrete proteins, which is closed related to the selection of effective transcriptional promoters and secretory signal peptides (Stephenson et al., 2011). Usp45 as the main signal peptide of *L. lactis*, which replaced the natural Nuc signal peptide, the efficiency of heterologous protein secretion has been greatly increased (Le Loi et al., 2001).

In addition to increasing the number of cellulolytic enzymes, in the future, the main focus in the construction of recombinant cellulolytic bacteria should be on improving the synergistic effect of designer cellulase system (Tarraran and Mazzoli, 2018). The complete degradation of rice straw was achieved by *Bacillus subtilis* SU40 β-glucosidase and *B. subtilis* MG7 endoglucosidase (Asha et al., 2015). Three different cellulase genes (*bg*, *eg*, and *cbh*) were co-expressed in *Saccharomyces cerevisiae*, and the enzyme activity on the filter paper was higher than each individual enzyme (Liu et al., 2018). The overexpression of *Nf*Bgl3A (thermophilic β-glucosidase from *Neosartorya fischeri*) by *Trichoderma reesei* (*T. reicheni*) significantly increased glucose yield and also improved the cellulose degradation performance of major cellulase components, including fibrinous disaccharide hydrolase and endoglucanase, compared with the addition of *Nf*Bgl3A to *T. reicheni in vitro* (Xue et al., 2016). β-glucosidase (*bglZ*) and endoglucanase (*eglII*) were expressed individually, fused (fus-*eglII* + *eglZ*), and co-expressed (coex-*bglZ* + *bglII*) in *B. megaterium* MS941, in the hydrolysis of bagasse. Compared with mixed-individual protein (mix-*eglII* + *bglZ*) and fus-*eglII* + *bglZ*, coex-*bglZ* + *eglII* resulted in faster and enhanced release of reducing sugar from bagasse (Kurniasih et al., 2014). In congo red staining with CMC-Na experiment, *L. lactis-cbh* did not have hydrolysis circles around them, and the supernatant and bacterial solution of *L. lactis-eg-cbh* showed only slight hydrolytic circles, but the hydrolytic circle after the concentration of its supernatant was not much different from that of the *L. lactis-eg* (Figure 4). It is speculated that the hydrolytic activity of supernatant after concentration may be due to the increase of EG content. In other words, the expression level of recombinant enzyme EG in *L. lactis-eg-cbh* is lower than that of *L. lactis-eg*. The same results can also be seen in Figure 5A, where recombinant enzyme CBH had no hydrolytic activity on CMC-Na, while EG-CBH had lower hydrolytic activity on CMC-Na than EG. Recombinant enzymes EG and CBH showed different specificity and hydrolytic activity for different substrates. The activity of crude enzyme solution obtained by *L. lactis-eg-cbh* against CMC-Na, filter paper and MCC were lower than that obtained by *L. lactis-eg*, higher than that by *L. lactis-cbh*. However, we found that EG-CBH produced by *L. lactis-eg-cbh* showed a synergistic effect on the degradation of RAC and cotton wool, and their enzyme activity were higher than EG produced by *L. lactis-eg-cbh*, even though the EG produced in *L. lactis-eg-cbh* might not be as much as *L. lactis-eg* (Figure 5A). This was consistent with the findings of Srisodsuk et al., CBHI of *Trichoderma reesei* alone was practically inactive toward cotton, and EGI of *Trichoderma reesei* slowly solubilized part of cotton. Working synergistically, EGI and the CBHI solubilized cotton more rapidly than EGI alone (Srisodsuk et al., 1998). Considering the activity of EG, CBH and EG-CBH enzymes and the synergistic effect of EG-CBH enzymes, RAC was determined as the substrate for the subsequent evaluation of enzyme activity.

Elevated temperature conditions offer manifold advantages, including heightened matrix solubility, augmented enzyme catalytic efficiency, and reduced contamination risks, so numerous industrial processes are conducted under high-temperature conditions (Schröder et al., 2014). Thermostable cellulases have emerged as pivotal candidates for industrial applications (huang et al., 2023). *Cbh* gene (from the *Caldicellulosiruptor bescii*) was expressed in *Escherichia coli* (BL21), its expression product CbCBH displayed peak activity against nitrobenzene cellobioside (pNPC) at 65°C and pH 6.0, and it demonstrated remarkable stability across a broad temperature range (60–80°C) for 8 h. CbCBH exhibited great thermostability making it a promising candidate for biofuel (Aqeel et al., 2024). In this study, the enzyme activity of the crude enzyme solution of recombinant strains increased with the increase in temperature. The optimum reaction temperature of the recombinant enzyme CBH was 60°C, while the recombinant enzymes EG and EG-CBH were tolerant to higher temperatures (80°C). This discovery indicated that the recombinant enzyme CBH, EG and EG-CBH holds promising potential for industrial applications, such as biofuel sector.

The lower the K_m_, the higher the binding affinity of the protease with a particular substrate was. K_cat_/K_m_ characterized the catalytic efficiency of different enzymes on specific substrates (Kaur et al., 2023). The determination of these parameters is important for understanding enzyme function, optimizing reaction conditions and developing new applications (McCarthy et al., 2003). In this study, Kinetic constants determined for recombinant enzymes in bacterial supernatant with RAC as substrate yielded similar K_m_, V_max_, K_cat_, and K_cat_/K_m_ values for *L. lactis*-cbh and *L. lactis*-eg-cbh. Bacterial supernatant of *L. lactis*-eg exhibited highest affinity for RAC (K_m_ value of 167.6 μmol∙L^−1^) and the highest catalytic efficiency (V_max_ value of 1,601 μmol∙min^−1^ mg^−1^, K_cat_ value of 73.09 s^−1^, K_cat_/K_m_ of 0.44 L∙s^−1^∙μmol^−1^) (Table 3). It is speculated that the activity of the recombinant enzyme EG-CBH produced by *L. lactis-eg-cbh* may be limited by the low activity of CBH in addition to the influence of the expression level of EG. Therefore, in order to improve the synergistic effect of EG-CBH enzyme expressed by recombinant bacteria, it is necessary to continue screening more potential CBH genes and optimize the co-expression system in the future.

Different metal ions have different affinity with amino acid residues of cellulase, which causes the conformational change of enzyme, thus stimulating or inhibiting the activity of enzyme. Fe^2+^, Co^2+^, and Mn^2+^ stimulate the activities of glucanase and xylanase in the rumen of goats (Cheng et al., 2016). Hg^2+^ could significantly reduce the activity of *Mycobacterium thermophilus* β-glucosidase (Pyeon et al., 2019). In this study, metal ions and chemical reagents affected the crude enzyme liquid of *L. lactis-eg-cbh* in a manner nearly similar to that of *L. lactis-eg*. Mn^2+^, Fe^2+^, Ca^2+^, Cu^2+^, and Co^2+^ promoted the expression of recombinant enzyme. While Zn^2+^, K^+^, and EDTA had little effect on the activity of recombinant enzyme, Hg^2+^ inhibited the activity of recombinant enzymes. The results of recombinant enzyme activity and assessment of enzymatic properties showed that the characteristics of *L. lactis-eg-cbh* were similar to those of *L. lactis-eg*, indicating the potential dominant role of endoglucanase in the whole co-expression process.

Liu et al. transferred *bgl1*, *cbh2*, and *egl3* genes from *T. reesei* into *L. lactis* MG1363, respectively, and obtained three engineered bacteria, which were mixed in equal proportions and added into high-moisture alfalfa silage. Compared to the control group (original lactic acid bacteria and lactic acid bacteria plus cellulase), silage showed a lower pH, ammonia nitrogen content, lack of butyric acid, and higher quality score (Liu et al., 2019). In order to evaluate the preliminary application effect of recombinants in silage fermentation, they were added to whole-plant corn silage in the laboratory. No significant differences were found between any of the assays in the groups treated with the addition of *L. lactis* NZ9000 to the whole-corn silage compared to the control group. The results indicated that the addition of *L. lactis* NZ9000 could not promote fermentation of the whole-corn silage directly. However, the use of recombinant *L. lactis-eg*, *L. lactis-cbh*, *L. lactis-eg* + *L. lactis-cbh*, *and L. lactis-eg-cbh* resulted in better silage potential, and especially contained recombinant bacteria that expressed the *eg* gene. For example, the pH and ADF and NDF contents of the silage in the *L. lactis-eg* group were the lowest among all the treatment groups, while the NH_3_-N contents of the silage in the *L. lactis-eg* + *L. lactis-cbh* group containing *L. lactis-eg* were lowest, together with the highest LA and CP contents. This could be attributed to the higher EG enzyme activity of the *L. lactis-eg*, leading to greater cellulose degradation and sugar production, and thus promoting lactic acid bacteria-mediated fermentation in the silage. Compared with *L. lactis-eg* group, *L. lactis-cbh* recombinantly expressing the *cbh* gene was less efficient, resulting in poorer silage quality, which might be related to the lower activity of cellobiose hydrolase produced by *L. lactis-cbh*. The *L. lactis-eg-cbh* group, in which the recombinant *eg* and *cbh* genes were co-expressed, showed higher pH, lower LA and WSC contents, and higher NDF and ADF contents of the silage compared to the *L. lactis-eg* + *L. lactis-cbh* group in which the two genes were expressed separately. This indicates that separate expression of the genes followed by mixing of the bacteria before addition to the silage resulted in better feed quality than when the genes were co-expressed. The above findings were consistent with the results of the congo red staining in the CMC-Na experiment and substrate specificity of recombinant enzymes experiment (Figures 4, 5A). It is speculated that the lower expression level of EG in *L. lactis-eg-cbh* affects its cellulose hydrolytic activity, providing further confirmation that the promotion of whole-corn silage fermentation by recombinant *L. lactis-eg* promoted was essentially dependent on the production of cellulose-degrading enzymes by the bacteria. Therefore, in order to enhance silage quality through the addition of recombinant lactic acid bacteria, it is imperative to meticulously select lactic acid bacteria with proven silage potential and proficient cellulase expression capabilities, thereby achieving a synergistic effect encompassing both lactic acid bacteria and cellulase.

## 5 Conclusion

Overall, the engineered strains *L. lactis-cbh*, *L. lactis-eg*, and *L. lactis-eg-cbh* were successfully constructed and EG, CBH, and EG-CBH were expressed. EG-CBH showed a synergistic effect on the degradation of RAC and cotton wool, and their enzyme activity were higher than EG and CBH. The enzyme activity of CBH reached its peak at 60°C and pH 6.0, while the optimum reaction conditions of EG and EG-CBH were 80°C and pH 6.0. EG exhibited higher affinity (K_m_ value of 167.6 μmol∙L^−1^) for RAC and the higher catalytic efficiency (K_cat_/K_m_ of 0.44 L∙s^−1^∙μmol^−1^) than CBH and EG-CBH, with no difference between the latter two. These attributes underscore its compelling potential across industrial cellulose degradation and production realms. In addition, Fe^2+^ and Ca^2+^ could be used to promote the enzyme activity of recombinase EG, Mn^2+^, Fe^2+^, Cu^2+^, and Co^2+^ can promote the enzyme activity of recombinase CBH, Fe^2+^, Ba^2+^ and higher concentrations of Ca^2+^, Cu^2+^, and Co^2+^ can promote activities of co-expression products of EG and CBH. The addition of engineered strains improved whole-corn silage fermentation and feed nutritional quality, with the best effect seen after addition of *L. lactis-eg*. One can thus conclude that the gene of lignocellulose-degrading enzyme from the yak rumenu microorganism and its engineering bacteria have important development prospects and application value in feed silage and are worthy of further development and research in the future.

## Data availability statement

The original contributions presented in the study are publicly available. This data can be found here: https://figshare.com/articles/online_resource/The_sequence_of_endoglucanase_and_cellobiohydrolase/26918479.

## Author contributions

XW: Conceptualization, Funding acquisition, Investigation, Methodology, Project administration, Resources, Validation, Visualization, Writing – original draft, Writing – review & editing. YS: Data curation, Software, Writing – original draft. YC: Formal analysis, Resources, Writing – review & editing. ZZ: Data curation, Investigation, Writing – original draft. HG: Data curation, Formal analysis, Writing – review & editing. YX: Data curation, Formal analysis, Writing – original draft. CW: Conceptualization, Funding acquisition, Methodology, Project administration, Resources, Supervision, Validation, Visualization, Writing – original draft, Writing – review & editing. YW: Conceptualization, Methodology, Project administration, Resources, Validation, Writing – review & editing. YY: Resources, Software, Writing – original draft.
